# Supplementary figures and images for: Analysis of deubiquitinase OTUD5 as a biomarker and therapeutic target for cervical cancer by bioinformatic analysis
Source: PeerJ. 2020 Jun 30;8:e9146. doi: 10.7717/peerj.9146 (PMC7333649; doi:10.7717/peerj.9146)

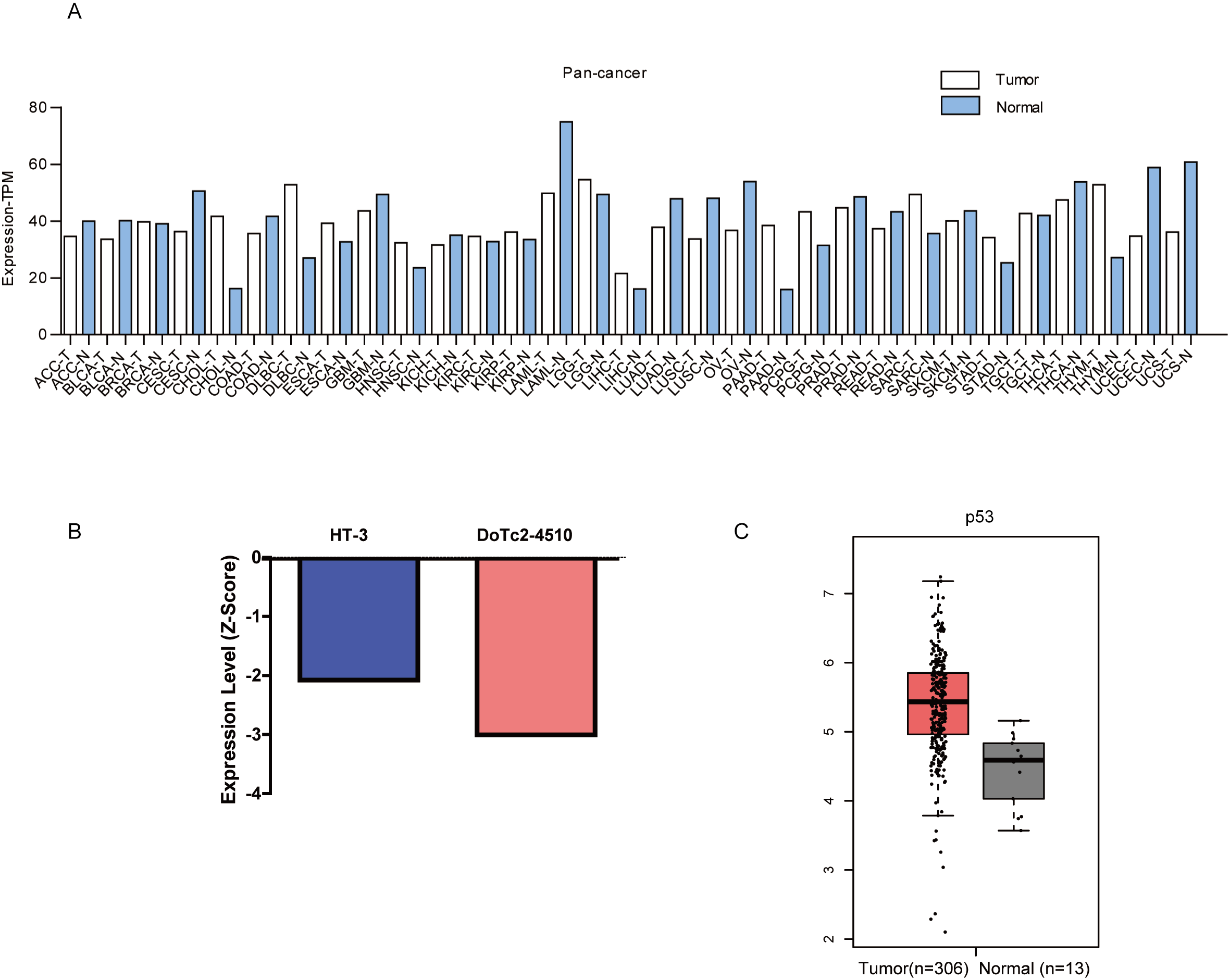

Supplement: Supplemental Information 1 — (A) Expression of OTUD5 in 31 types of normal and tumor samples. Transcripts per million (TPM) of OTUD5 in CESC (TCGA) from the GEPIA database. (B) Expression of OTUD5 in cervical cancer cell lines (HT-3 and DoTc2-4510) through COSMIC database analysis. (C) Box plot showing the transcripts per million (TPM) of p53 in CESC (TCGA) from the GEPIA database. [file peerj-08-9146-s001.png]
